# Structural Changes of β-Casein Induced by Temperature and pH Analysed by Nuclear Magnetic Resonance, Fourier-Transform Infrared Spectroscopy, and Chemometrics

**DOI:** 10.3390/molecules26247650

**Published:** 2021-12-17

**Authors:** Tatijana Markoska, Davor Daniloski, Todor Vasiljevic, Thom Huppertz

**Affiliations:** 1 Advanced Food Systems Research Unit, Institute for Sustainable Industries & Liveable Cities, College of Health and Biomedicine, Victoria University, Melbourne, VIC 8001, Australia; tatijana.markoska@live.vu.edu.au (T.M.); davor.daniloski@live.vu.edu.au (D.D.); todor.vasiljevic@vu.edu.au (T.V.); 2 Teagasc Food Research Centre, Food Chemistry and Technology Department, Moorepark, Fermoy, P61 C996 Cork, Ireland; 3 FrieslandCampina, 3818 LE Amersfoort, The Netherlands; 4 Food Quality and Design Group, Wageningen University & Research, 6808 WG Wageningen, The Netherlands

**Keywords:** FTIR, NMR, β-casein, secondary structure, temperature, pH

## Abstract

This study investigated structural changes in β-casein as a function of temperature (4 and 20 °C) and pH (5.9 and 7.0). For this purpose, nuclear magnetic resonance (NMR) and Fourier-transform infrared (FTIR) spectroscopy were used, in conjunction with chemometric analysis. Both temperature and pH had strongly affected the secondary structure of β-casein, with most affected regions involving random coils and α-helical structures. The α-helical structures showed great pH sensitivity by decreasing at 20 °C and diminishing completely at 4 °C when pH was increased from 5.9 to 7.0. The decrease in α-helix was likely related to the greater presence of random coils at pH 7.0, which was not observed at pH 5.9 at either temperature. The changes in secondary structure components were linked to decreased hydrophobic interactions at lower temperature and increasing pH. The most prominent change of the α-helix took place when the pH was adjusted to 7.0 and the temperature set at 4 °C, which confirms the disruption of the hydrogen bonds and weakening of hydrophobic interactions in the system. The findings can assist in establishing the structural behaviour of the β-casein under conditions that apply as important for solubility and production of β-casein.

## 1. Introduction

Caseins represent the major protein constituents of the bovine milk (80%), comprised of four major phosphoproteins, α_S1_-, α_S2_-, β-, and κ-casein at concentration of 12–15, 3–4, 9–11 and 2–4 g/L, respectively [1]. In the dairy industry, the structural organisation of caseins can have a great effect on the functional properties of milk and the quality of the final product due to various interactions (hydrophobic, electrostatic, van der Waals forces, or covalent bonds) yielding to different properties. As the most amphiphilic, bovine β-casein represents 35% of the caseins in the bovine milk with 209 amino acids in the polypeptide chain and average molecular weight of 24 kDa [2]. Due to the presence of many relatively hydrophobic parts in the β-casein molecule with great number of Pro residues, the molecule can adopt flexible conformations. These conformations are characterised by large range of inter or intra molecular interactions, fewer secondary and tertiary structures, while containing more random coil organisations [3]. 

There have been multiple studies analysing the secondary structure of β-casein. The summarised findings included the presence of 7–25% α-helical structures, 15–33% β-sheets, 20–30% turns, and 20–25% polyproline II structures [2,4,5,6]. The predicted α-helical structures are likely in the N-terminal part of the β-casein, f(1–40), due to the presence of phosphoserine residues that carry the net charge of the molecule [7]. The apolar residues present in the C-terminal part of β-casein, f(136–209), are responsible for the appearance of β-sheet structures due to higher hydrophobicity in this region [7,8]. 

Temperature is an important factor in regards to the β-casein release from the casein micelle. In a cold environment, due to weakening of hydrophobic attraction, selective solubilisation of β-casein takes place, leading to increased solubility of β-casein during cold storage [9]. At low temperature, β-casein appears as monomer with structural transitions due to cold denaturation. The dissociation of β-casein from the casein micelle was previously observed to be the greatest at pH 5 and a temperature below 4 °C [10]. Moreover, the net negative charge at high pH increases as a result of deprotonation of carboxyl groups and loss of the positive charge of amino acids including His. The increase in electrostatic repulsion takes place as a result of the negative charge of the phosphoseryl residues [11]. The transition that takes place on the β-casein molecule based on pH and temperature adjustment can have a significant effect on the secondary structure of the molecule. The impact of both pH and temperature on β-casein and overall milk protein structure play an important role in protein stability during processing of milk and milk products and their storage. The pH of dairy products may vary from <4 for some fermented dairy to >7 for some dairy ingredients and infant formula products, whereas storage occurs both at ~4 °C for pasteurised products and at room temperature for sterilised products. 

Even though several studies already confirmed the presence of some structural elements in the β-casein molecule, it would be valuable to know how this particular casein adapts its secondary structural elements in different environments [4,12,13]. Thus, the aim of the present research was to identify the structural components of β-casein using Fourier-transform infrared spectroscopy (FTIR) and nuclear magnetic resonance (NMR). FTIR spectroscopy provides information of the structural changes in proteins by absorption of the stretching/bending vibrations in different regions [14]. Consequently, NMR provides detailed information of the protons in the molecule, which evaluates the primary position of the atoms in the molecule [15]. Both methods are nondestructive with minimal sample preparation and can provide information under any condition. The current work involved structural analysis of β-casein at two pH values (pH 5.9 and pH 7.0) and two temperatures (4 and 20 °C) in order to establish the structural transitions of β-casein. The selected temperature was used to observe the structural transition of β-casein in most used storage conditions for milk proteins. The pH 7.0 was used to observe how β-casein structure changes in pH environment close to native pH in milk and pH 5.9 were used as the native pH of synthesised β-casein and also were important for curd stability during milk coagulation. 

## 2. Results and Discussion

In the current study, FTIR and NMR were used to examine the structural changes in β-casein resulting from changes in pH and temperature. In the Amide I region of the FTIR spectra, six structural features were distinguished and analysed (Table 1).

From the peak area calculations of the second derivative spectra, the most significant differences (*p* ≤ 0.05) as a result of changes in pH and temperature were observed in random coil, α-helix, and β-turn structures. At pH 7.0, the percentage of random coil structure was ~15% higher at 4 °C than at 20 °C, whereas random coil structures were not detected at pH 5.9 at either temperature. The assigned random coil structures have previously been shown to include short polyproline II (PPII) helix/chains [16]. Bovine β-casein contains 35 Pro residues, evenly distributed along the 209 amino acid polypeptide chain, which favours the formation of PPII structures [4]. The lack of detected random coil structures at pH 5.9 indicates that there was no substantial unfolding of the secondary structure of the protein at this pH [17]. The absence of random coils in β-casein at pH 5.9 appeared to correlate with a greater amount of α-helical structures, at both 4 and 20 °C. At 20 °C, the α-helix content at pH 7.0 was half of that observed at pH 5.9, whereas α-helical structures were not detected at pH 7.0 at 4 °C. The β-turn structures were affected by temperature, but not by pH; at both pH 5.9 and 7.0, fewer β-turns were observed at 20 than at 4 °C. Farrell et al. [4] stated that with the temperature increase the conversion from β-turns and β-sheet to PPII conformations might occur. Hence, in line with these previous findings in combination with the present data of random and turn conformations (Table 1), there is a reasonable possibility of transformation of these structures into PPII helices, whose content was found to be between 20 and 25% within the β-casein molecule [5,6]. The formation of β-turns in caseins is promoted by the Pro residues by cross-linking with neighbouring residues via van der Waals interactions acting as a β-turn inducer [8]. 

**Figure 1:**
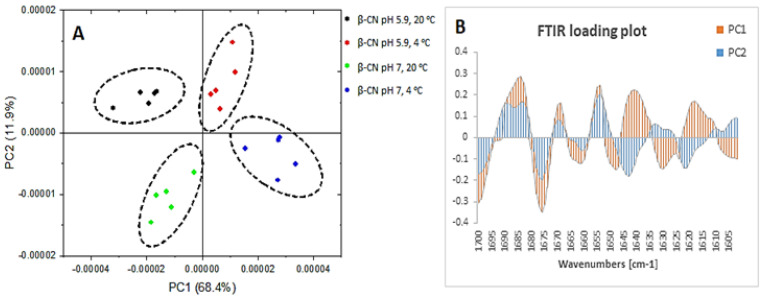
Principal components score plot (**A**) and loading plot (**B**) of β-casein of the FTIR spectra in region 1700–1600 cm^−1^ where β-casein at pH 5.9 and temperature of 20 °C (black), β-casein at pH 5.9 and temperature of 4 °C (red), β-casein at pH 7.0 and temperature of 20 °C (green) and β-casein at pH 7.0 and temperature of 4 °C (blue) are in (**A**) and PC1 (orange) and PC2 (blue) are in (**B**).

The observed differences in the peak area (Table 1) were further confirmed by PCA results (Figure 1).

In addition, PC1 confirmed 68.4% difference between the samples at 4 and 20 °C, while PC2 confirmed 11.9% difference between samples at pH 5.9 and pH 7.0 (Figure 1A). The loading score in Figure 1A visually separated the groupings of samples that differed in temperature along the PC1. The PC1 also distinguished the differences in the structural components between these samples, which are confirmed in the loading plot in Figure 1B. The PC1 identified high loading for a peak at 1650 cm^−1^ (α-helix) for samples at 20 °C, confirming a greater presence of this secondary structure at this temperature. On the other hand, the observed high loading for peak at 1640 cm^−1^ for samples at 4 °C confirmed the high level of random structures. PC1 also depicted slight changes in β-sheets by greater presence of intramolecular β-sheets (1630 cm^−1^) for samples at 20 °C and greater intermolecular β-sheets (1620 cm^−1^) for samples at 4 °C. Similarly, PC2 differentiated the structural features occurring between samples with different pH values which were also present with different loadings in the loading plot in Figure 1B. In the PC2 loading plot (Figure 1B), great loadings were observed for a peak at 1653 cm^−1^ (α-helix) for samples at pH 5.9 and at 1643 cm^−1^ (random coils) for samples at pH 7.0. Regarding the β-sheet structures, greater loading for a peak at 1630 cm^−1^ (intramolecular β-sheets) was observed for samples at pH 5.9 and at pH 7.0 greater loading shifted to indicate intermolecular β-sheets (peak 1623 cm^−1^). Thus, PC1 and PC2 successfully confirmed the previously observed changes for α-helix and random structures and additionally confirmed differences in inter- and intramolecular β-sheets.

**Figure 2:**
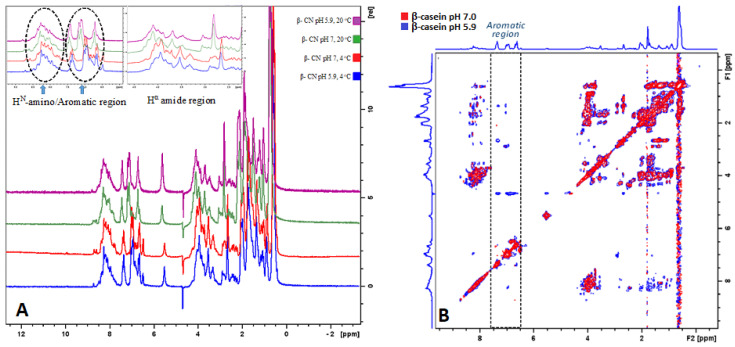
(**A**) Overlayed ^1^H NMR spectra of β-casein at pH 5.9 and temperature of 20 °C (purple), β-casein at pH 5.9 and temperature of 4 °C (blue), β-casein at pH 7.0 and temperature of 20 °C (green) and β-casein at pH 7.0 and temperature of 4 °C (red). (**B**) Overlayed TOCSY NMR spectra of β-casein at pH 7.0 (red) and β-casein at pH 5.9 (blue).

The ^1^H-NMR chemical shifts in proteins are sensitive to local structural rearrangements. Moreover, the conformational differences can be a result of multiple contributions including torsion angles coming from backbone and side chains, hydrogen bonding, electric fields, rings vibrations, and steric repulsions [18]. In the current work, we observed the variations in chemical shift distribution in four regions including methyl -CH_3_- (0–1.5 ppm), aliphatic -CH_2_- (1.5–3.5 ppm), H^α^ amide region (3.5–5 ppm), and aromatic/H^N^-amino region (5.5–10 ppm). From the overlayed spectra in Figure 2A, a downfield shift of the proton chemical shifts can be seen at 4 °C compared to at 20 °C. In addition, the deshielding was observed in all the regions, except in the amino or backbone region (Figure 2A). Thus, in the amino region the peaks appeared in a broad pattern which overlayed in identical chemical shift positions (Figure 2A). 

The deshielding in the other regions resulted by lowering of temperature to 4 °C was observed to be 0.2–0.3 ppm. The sensitivity of the ^1^H chemical shift can be promoted by multiple factors, including the protein sensitivity to conformations, hydrogen bonding, ring vibrations, or electric fields [19]. In addition, the upfield and downfield shielding of the ^1^H chemical shift can result from changes in the different components in the secondary structure [18,20]. In this study, an opposite effect by 0.3 ppm downfield shift for the amide region was observed, which can relate to a lower presence of α-helical structures at low temperature. In addition, from the integrated amide region we observed a significant decrease (≤0.05) in the peak intensities by ~3% at 4 °C compared to at 20 °C (Table 2). This further confirms previously discussed FTIR results (Figure 1 and Table 1) regarding the absence or decline in α-helical structures at low temperature.

From the other regions of the NMR spectrum a greater amount of methyl groups at low temperature was observed, which was more present at pH 5.9 (Figure 2A). The peak observed in this region (0–0.8 ppm) likely appeared from the side chains (CH_3_) groups from Ile, Val, and Leu in β-casein [21]. The lower peak intensity for methyl groups at room temperature can be due losses of signal from the methyl side chains of Ile, Val, and Leu. An increase in pH from 5.9 to 7.0 led to slightly lower peaks for the methyl side chains (Table 2). The amide region of the β-casein presented slightly greater intensity at 20 °C compared to that at 4 °C at both pH values (Table 2). 

The change in pH from 5.9 to 7.0 lead to lower detection of the cross peaks in the aromatic region of the TOCSY spectra (Figure 2B). This spectral pattern was not affected by the temperature, i.e., at both 4 and 20 °C the TOCSY spectra of β-casein showed the same pattern in regard to the pH. In the aromatic region of the TOCSY spectra the cross peaks present the interactions of the proton rings with the neighbouring protons of the amino acids [15]. It was previously suggested that charge-induced disruption of the cohesive interactions in the hydrophobic regions of the caseins occur as a result of pH-induced charge modifications on side groups of amino acids, including Tyr, Lys, His, and Arg [22]. In the pH range currently studied, His would be the most susceptible to (de)protonation as a result of pH change. The side groups of these amino acids are detected in the aromatic region of the TOCSY spectra and the cross peaks in this region denote their connection with the neighbouring protons. It is known that increase in pH leads to greater intermolecular repulsion of caseins [11]. The addition of alkali to the β-casein solution can lead to disruption of existing hydrophobic connections, which was observed by reduction of the proton–proton cross association in the TOCSY spectra. 

**Figure 3:**
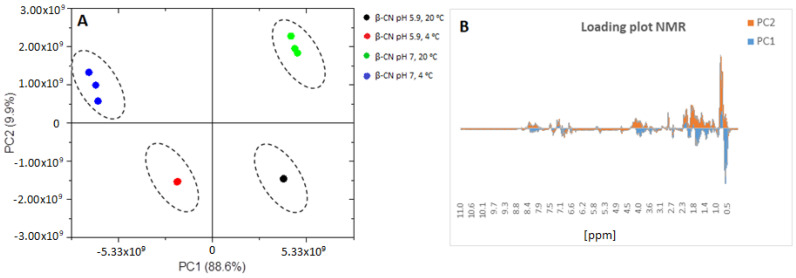
Principal components score plot (**A**) and loading plot (**B**) of β-casein of the NMR spectra in where β-casein at pH 5.9 and temperature of 20 °C (black), β-casein at pH 5.9 and temperature of 4 °C (red), β-casein at pH 7.0 and temperature of 20 °C (green) and β-casein at pH 7.0 and temperature of 4 °C (blue) are in (**A**) and PC1 (blue) and PC2 (orange) are in (**B**).

The observed structural differences resulted from changes in temperature and pH were also evaluated by PCA analysis (Figure 3).

In the PCA loading score (Figure 3A), PC1 separated the results based on the temperature with 88.6% variance. On the other hand, PC2 separated the β-casein based on pH effect with 9.9% variance. PC1 has shown greater loading for a peak at 0.5 ppm for samples at low temperature and PC2 indicated that this peak was mainly present for samples at pH 7.0. Moreover, PC2 showed that peak loadings in aliphatic (1.5–3.5 ppm) and amide regions (3.5–5.0 ppm) were more prominent for samples at pH 7.0, whereas at pH 5.9 greater loading was observed for the aromatic region (6.5–8.0 ppm). Furthermore, both PC1 and PC2 confirmed that in the aliphatic and aromatic regions the peak loading was more intense for samples at pH 7.0 and temperature of 4 °C, which can confirm the structural changes observed by FTIR occurring as a result of a cold environment and pH.

## 3. Material and Methods

### 3.1. Sample Preparation

Bovine β-casein was purchased from Sigma-Aldrich (Sigma-Aldrich, St. Louis, MO, USA) with >98% purity. The preparation process was performed by dispersion of 10 mg β-casein in 1 mL of a mixture of H_2_O:D_2_O (90%:10%, Sigma-Aldrich, St. Louis, MO, USA) for both FTIR and NMR analyses. The pH of β-casein in H_2_O/D_2_O solution was measured using a pH meter (Metrohm AG, Herisau, Switzerland) equipped with a combined pH electrode with temperature sensor and fixed cable. The original pH of the protein solution was 5.9. The pH was further adjusted to 7.0 using 0.25 M NaOH. The pH adjustment was carried out under continuous stirring of the solution and pH was additionally controlled before the structural measurements. Both β-casein solutions with pH 5.9 and pH 7.0 were analysed for structural features at 4 and 20°C. The temperature for the FTIR analysis was monitored with temperature probe, however for the NMR analysis the temperature control was automated by the instrument.

### 3.2. Fourier Transform Infrared Spectroscopy

The secondary structure of β-casein as a function of pH and temperature was analysed using a FTIR spectrometer (Frontier, PerkinElmer, Boston, MA, USA) in the range of 4000 to 600 cm^−1^ with a resolution of 4 cm^−1^ and 16 scans for each spectrum. Before the start of the measurement for every sample, the background spectra were scanned using a blank diamond attenuated total reflectance (ATR) cell. At the start of the analysis the solvent spectrum (90% H_2_O/10% D_2_O) was recorded and used for subtraction from the sample spectra to eliminate the intense solvent signal. The spectra of five subsamples of each sample were taken by refilling the ATR cell. For the secondary structure analysis, the amide I region (1700–1600 cm^−1^) was analysed for C=O stretching after applying Savitzky–Golay smoothing and 2nd derivative. The FTIR spectra and mean centering were analysed using Spectragryph software (version 1.2.7, Oberstdorf, Germany). The baseline of five repetitions for each sample was subtracted and the area of the peak was analysed by Origin software (Origin Pro 2021, v. 95E, OriginLab Corporation, Northampton, MA, USA). To estimate the area of each component representing secondary structures, a technique previously described elsewhere [23] was utilised. Briefly, each spectrum from five repetitions was treated by interpolation baseline mode and adjacent-averaging smoothing, then each peak in the 2nd derivative spectra was selected and integrated by high percentage. In the amide I region several peaks were analysed, including side chain (1608–1610 cm^−1^), β-sheets (1620–1630 cm^−1^), random coil (1640–1645 cm^−1^), α-helix (1646–1652 cm^−1^), β-turns (1677–1679 cm^−1^) and aggregated β-sheets (1689–1690 cm^−1^) [24].

### 3.3. Nuclear Magnetic Resonance

The NMR analysis was performed on a Bruker Avance spectrometer (Bruker BioSpin GmbH, Rheinstetten, Germany) operating at a 600 MHz transmitter frequency using 5 mm TXI probe with z- gradient. Proton NMR (^1^H-NMR) spectra were acquired using 16 scans and spectral width of 9615 Hz in three replications. The two-dimensional methods used in this study were total correlation spectroscopy (TOCSY) and nuclear Overhauser effect spectroscopy (NOESY). The 2D spectra were recorded using 8 scans and spectral width of 8196 Hz for TOCSY and 5882 Hz for NOESY spectra. The water suppression for spectra was performed using excitation sculpting with gradients with acquisition mode of States-TPPI (time-proportional phase incrementation). All spectral data were processed using TopSpin (version 4.0.6) software (Bruker BioSpin). The phase correction was performed manually by either 0th or 1st order correction for pk or the baseline and the noise correction was adjusted using qfil mode to 0.1 ppm filter width and symmetrical noise correction for the homonuclear spectra. The NMR spectra were analysed using three NMR regions, i.e., amino/aromatic (H^N^), amide (H^α^), and aliphatic. From the aliphatic region, the methyl peak (CH_3_) was analysed separately due to its high loading. All the peaks observed in these regions were manually integrated in TopSpin (version 4.0.6) software (Bruker BioSpin). 

### 3.4. Statistical Analysis and Spectral Data

To assess the experimental results, Minitab version 20 software was used to analyse the data (Minitab Inc., State College, PA, USA). Hence, a two-way ANOVA and Tukey’s test were performed with β-casein as fixed factor to evaluate if there was a difference between means (*p* ≤ 0.05). Unless otherwise specified, all measurements were taken in triplicate. In addition, principal component analysis (PCA) was used for both FTIR and NMR results to identify the changes in the secondary structure in β-casein samples based on the temperature and pH effect. PCA gives information by generating principal components (PCs) as a coordinated axis with a least possible loss of information. The groupings of the different samples were depicted in score plots and the loading plots were used for the wavenumbers responsible for peaks classification. The multivariate analysis was carried out at a 95% confidence level. 

## 4. Conclusions

The combination of FTIR and NMR was very efficient in characterisation of structural differences in the β-casein molecule as affected by temperature and pH. Moreover, both methods confirmed similar structural transitions of the β-casein molecule in regard to the applied conditions, indicating greater structural changes taking place as a result of the temperature. A temperature of 4 °C is critical for β-casein molecules due to lowering of hydrophobicity that results in unfolding and opening up of the native conformers and liberalisation of the monomers from the casein micelle. The restructuring of the β-casein was confirmed by FTIR results implying formation of more random coils at 4 °C at the expense of the α-helical structure. Moreover, this was intensified by changing the pH to 7.0. Hence, the side chains of amino acids known as α-helix inducers showed lower detection due to loss of hydrogen bonding resulting in reduction of the α-helix in the β-casein polypeptide chain. The observed structural changes promoted by the temperature change were more intense at high pH, however at pH 5.9 the structural reorganisation was minor. The current findings confirmed that both pH and temperature have a great effect on structure of a β-casein molecule. Moreover, the results can assist in understanding the behavior of β-casein during β-casein production, solubility, processing of milk and milk products, and storage. In addition, FTIR and NMR have proven to successfully detect structural transition in β-casein. The combination of these methods can be further expanded to other proteins and assist in understanding protein behavior during food processing. 

## Figures and Tables

**Table 1:** Total percentage areas of different secondary structures in Amide I in β-CN in FTIR including side chain, β-sheet, random coil, α-helix β-turn, and aggregated β-sheets. The selected band frequency for each structural component is presented in cm^−1^. The peak area percentage is presented for temperature of 4 and 20 °C and pH of 5.9 and 7.0.

Band Assessment	Band Frequency (cm^−1^)	Peak Area (%)			
		Temperature 4 °C		Temperature 20 °C	
		pH 5.9	pH 7.0	pH 5.9	pH 7.0
Side chain	1608–1610	3.99 ± 0.53 ^a^	5.40 ± 0.09 ^a^	3.92 ± 0.34 ^a^	4.33 ± 0.60 ^a^
β-sheet	1620–1630	26.04 ± 3.55 ^a^	23.07 ± 4.24 ^a^	26.70 ± 4.03 ^a^	23.72 ± 2.47 ^a^
Random coil	1640–1645	n/d	46.53 ± 5.81 ^b^	n/d	31.07 ± 8.71 ^a^
α-helix	1646–1652	42.98 ± 3.41 ^b^	n/d	45.24 ± 3.76 ^b^	21.77 ± 2.59 ^a^
β-turn	1677–1679	22.25 ± 1.87 ^c^	20.04 ± 1.35 ^bc^	17.00 ± 2.29 ^ab^	13.34 ± 4.66 ^a^
Aggregated β-sheet	1689–1690	4.75 ± 1.61 ^a^	4.96 ± 0.97 ^a^	7.15 ± 1.36 ^a^	5.78 ± 2.02 ^a^

^a,b,c^ Mean values within a row that do not share a common superscript letter are significantly different (*p* ≤ 0.05); n/d = not detected.

**Table 2:** Total percentage areas peaks in different regions for β-casein in NMR including methyl, aliphatic, amide and amino region. The difference in the percentage is presented for β-casein at temperature of 4 and 20 °C and pH of 5.9 and 7.0.

Temperature	4 °C		20 °C	
pH	5.9	7.0	5.9	7.0
Region/Integral	β-Casein			
Methyl	19.48 ± 0.16 ^a^	17.63 ± 0.27 ^b^	16.78 ± 0.13 ^bc^	16.22 ± 0.17 ^c^
Aliphatic	16.22 ± 0.16 ^ab^	17.73 ± 0.75 ^a^	14.91 ± 1.64 ^b^	13.90 ± 0.35 ^bc^
Amide	42.29 ± 0.04 ^b^	42.82 ± 0.21 ^b^	46.69 ± 0.14 ^a^	46.32 ± 0.17 ^a^
Amino	22.01 ± 0.34 ^b^	21.83 ± 0.82 ^b^	22.62 ± 0.41 ^ab^	23.47 ± 0.89 ^a^

^a,b,c^ Mean values within a row that do not share a common superscript letter are significantly different (*p* ≤ 0.05).

## Data Availability

Data are contained within the article.

## Sample Availability

Samples of the compounds are not available from the authors.
